# Innovation in Education Across the Care Continuum

**DOI:** 10.1093/geroni/igaf122.1355

**Published:** 2025-12-31

**Authors:** Kristen Neises

## Abstract

The Geriatric Academic Career Award (GACA) has been a critical catalyst for advancing geriatrics education, developing future healthcare professionals, and addressing the growing needs of an aging population. GACA-supported scholars have led initiatives that integrate geriatrics into medical, nursing, and allied health curricula, expand workforce training, and improve care for underserved older adults. These efforts have enhanced clinical training, developed novel educational methodologies, and strengthened interprofessional collaboration, ensuring that learners are prepared to deliver high-quality care to older adults across various healthcare settings. Despite its success, sustaining these innovations remains a challenge due to a lack of structured, long-term funding pathways. In this symposium, six GACA recipients will showcase the innovative educational programs they have developed, highlighting how their work has transformed workforce training and improved health outcomes for older adults. Presenters will discuss re-envisioning geriatrics education in nursing using oral history methodology, addressing care gaps for aging homeless populations, developing interdisciplinary training models, and creating geriatrics curricula for nursing home-based education for nursing and medicine. By reflecting on the successes and challenges of implementing and sustaining their programs, panelists will illustrate the need for continued investment in geriatrics education and stronger collaboration between clinicians, educators, and researchers. This session will explore emerging strategies to embed geriatrics education across disciplines and settings, ensuring that innovations in training continue to shape the future of aging-related care.

